# Depression and Suicide Risk Screening in the Veterans Health Administration

**DOI:** 10.1001/jamanetworkopen.2024.51936

**Published:** 2024-12-23

**Authors:** Lucinda B. Leung, Karen Chu, Martin L. Lee, Amy G. Bonilla, Edward P. Post, John C. Fortney

**Affiliations:** 1 Center for the Study of Healthcare Innovation, Implementation, and Policy, VA Greater Los Angeles Healthcare System, Los Angeles, California; 2 Division of General Internal Medicine and Health Services Research, Department of Medicine, University of California, Los Angeles David Geffen School of Medicine; 3 Department of Biostatistics, University of California, Los Angeles Fielding School of Public Health; 4 Center for Clinical Management Research, VA Ann Arbor, Ann Arbor, Michigan; 5 Department of Medicine, University of Michigan Medical School, Ann Arbor; 6 Department of Psychiatry and Behavioral Sciences, School of Medicine, University of Washington, Seattle; 7 Seattle-Denver Center of Innovation for Veteran-Centered and Value-Driven Care, Seattle, Washington

## Abstract

This cohort study investigates rates and results of depression and suicide risk screening in the Veterans Health Administration from 2018 to 2024.

## Introduction

Many speculate that the COVID-19 pandemic worsened veteran mental health. The Veterans Health Administration (VHA) has near-universal depression screening, closely monitors for new depressive episodes, and aims to provide appropriate follow-up and treatment.^1,2^ Beginning in 2018, the VHA became the largest integrated health care system aiming to screen universally for suicide risk.^3^ This study aimed to identify trends in depression and suicide risk screening among veterans.

## Methods

This 5-year cohort study with an interrupted time series analysis examined receipt and results of mental health screening for VHA patients. Patients without preexisting depression diagnoses received the Patient Health Questionnaire-2 (PHQ-2) annually. Patients were screened for suicide risk with PHQ-9 item 9 and then Columbia Suicide Severity Risk Scale (C-SSRS), simplified to only C-SSRS in 2020. PHQ-2 and item 9 or C-SSRS screens were counted once per year, with positive screenings noted if there were multiple screenings. Regression models estimated monthly rates of screens administered and new positive screens (PHQ-2 score ≥3 for depression and “yes” on C-SSRS items 3, 4, 5, or 6b for suicide risk) nationally during 3 periods (before [October 1, 2018, to February 30, 2020], early [March 1, 2020, to September 30, 2021], and late [October 1, 2021, to September 30, 2023] COVID-19 pandemic). Models included fixed effects for screening location to account for time-invariant facility characteristics (eg, rurality and case mix). Conducted October 2, 2023, to January 18, 2024, this study was determined exempt from review and informed consent by the VA Greater Los Angeles Healthcare System Institutional Review Board because it analyzed preexisting data; it followed the STROBE reporting guideline.

## Results

**Figure 1.**
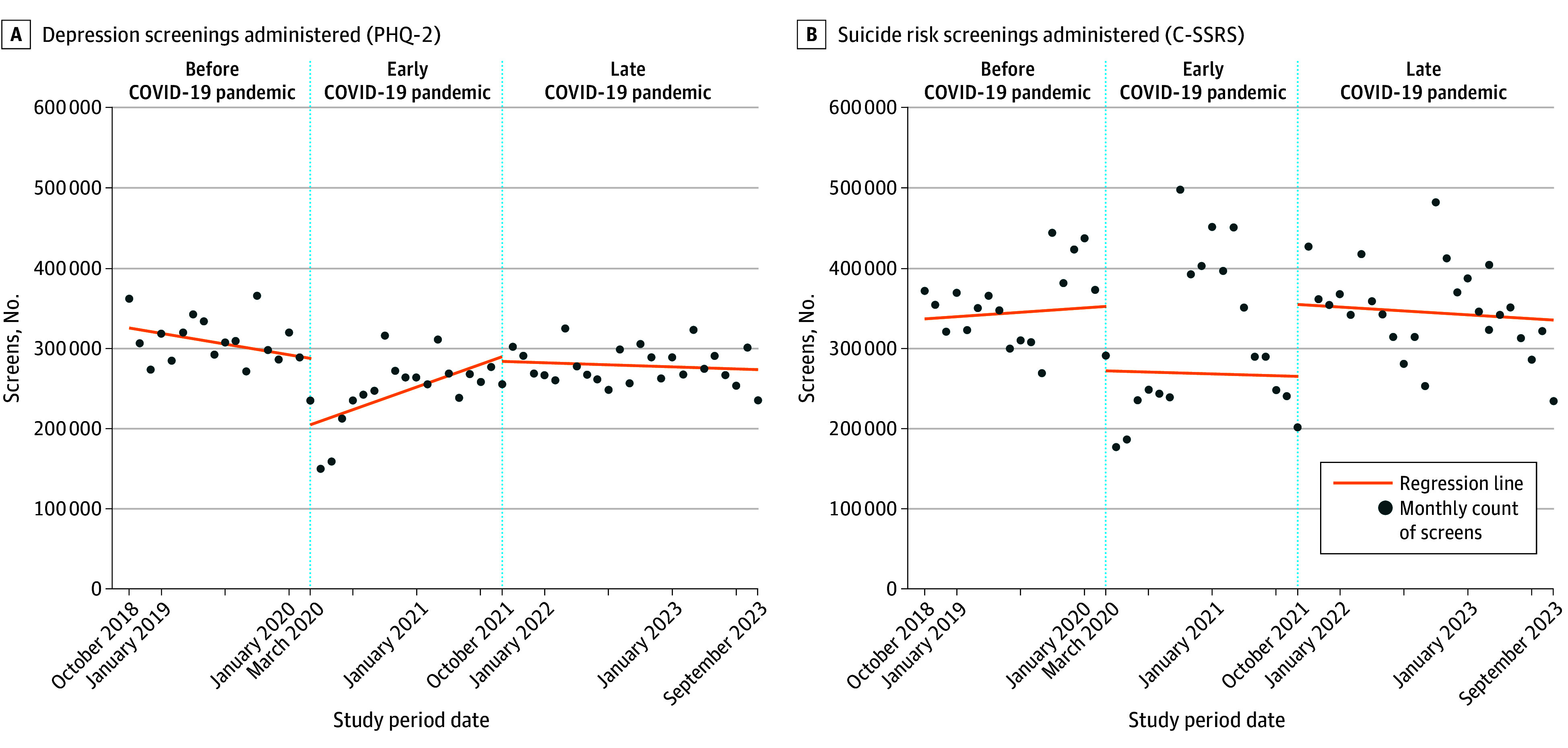
Depression and Suicide Risk Screens Administered Over Time This interrupted time series analysis estimated monthly counts of screens administered using the Patient Health Questionnaire-2 (PHQ-2) and Columbia Suicide Severity Risk Scale (C-SSRS) nationally during 3 periods (before [October 1, 2018, to January 30, 2020], early [February 1, 2020, to September 30, 2021], and late [October 1, 2021, to September 30, 2023] COVID-19 pandemic). Logistic regression models included fixed effects for where screening occurred to account for time-invariant facility characteristics. For all models, significance was determined using a 2-tailed α = .05. All analyses were conducted using Stata/MP version 18 (StataCorp).

**Figure 2.**
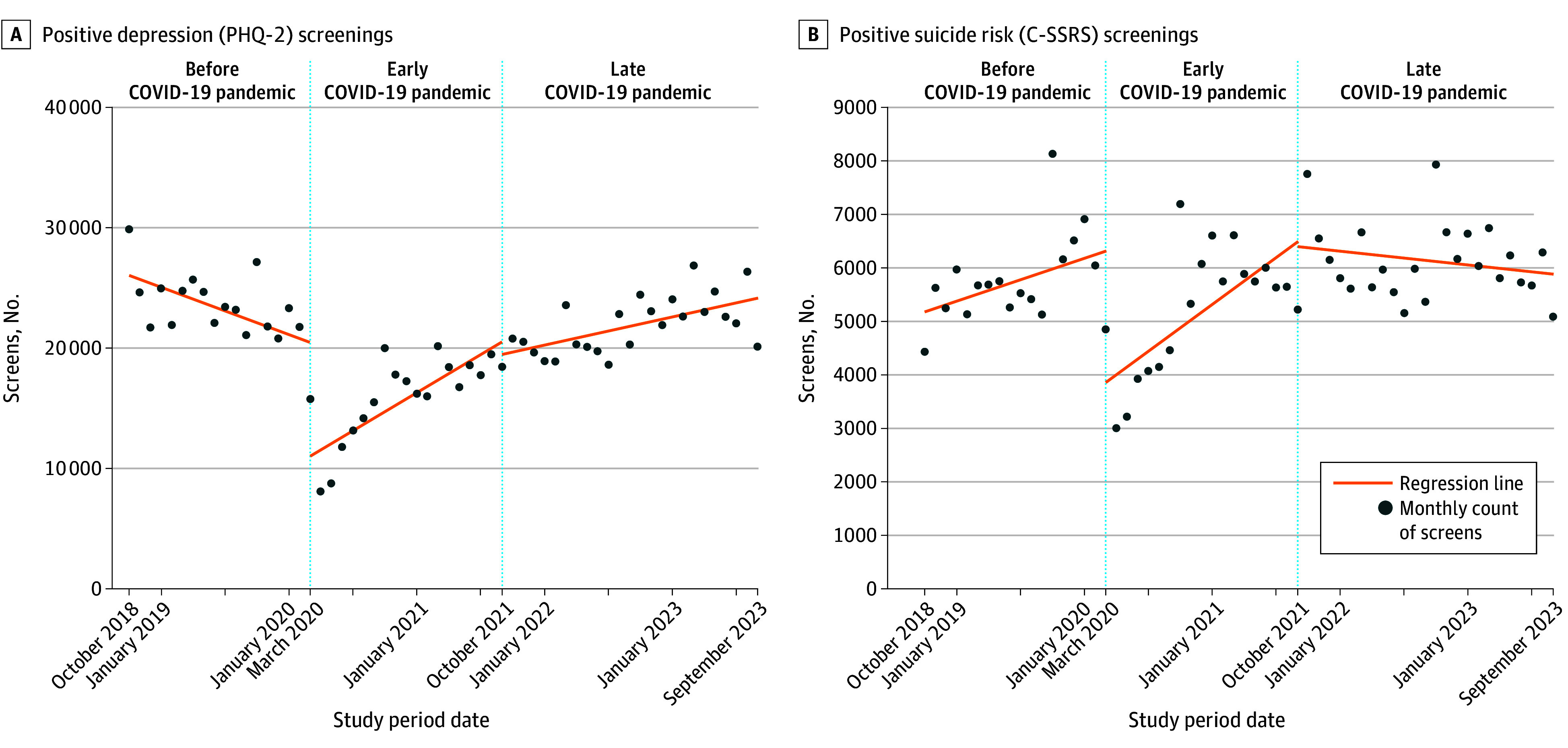
Positive Depression and Suicide Risk Screens Over Time This interrupted time series analysis estimated monthly counts of new positive screens (Patient Health Questionnaire-2 [PHQ-2] score ≥3 for depression and “yes” on Columbia Suicide Severity Risk Scale [C-SSRS] items 3, 4, 5, or 6b for suicide risk) nationally during 3 periods (before [October 1, 2018, to January 30, 2020], early [February 1, 2020, to September 30, 2021], and late [October 1, 2021, to September 30, 2023] COVID-19 pandemic). Logistic regression models included fixed effects for where screening occurred to account for time-invariant facility characteristics. For all models, significance was determined using a 2-tailed α = .05.

Among 9 191 729 VHA patients analyzed (mean [SD] age, 60 [19] years; 7 657 826 males [83.3%]; 230 486 873 screening encounter months), a mean of 278 668 PHQ-2 and 337 470 C-SSRS screenings were administered monthly; 7.4% and 1.7% were positive, respectively, and required clinician follow-up. Interrupted time series models revealed a 0.7% increase per month in odds of depression screening during the early pandemic (*P* < .001) and no change (odds ratio, 1.002; 95% CI, 0.9998-1.005) during the late pandemic (*P* = .07) compared with before the pandemic (Figure 1). Conversely, there was a 1.6% and 1.9% reduction per month in odds of suicide risk screening during early and late pandemic periods, respectively (all *P* < .001). The COVID-19 pandemic was associated with a small but significant 2.1% and 1.9% increase per month in odds of newly positive depression screens during early and late periods, respectively (all *P* < .001), compared with prepandemic rates (Figure 2). We also observed a 1.4% increase per month in odds of newly positive suicide risk screens during the early (*P* < .001) but no change during the late COVID-19 period.

## Discussion

This cohort study’s findings provide evidence of a small but significant worsening of depressive symptoms and suicide risk (early–COVID-19 period only) among US veterans. Our findings align with previous research on the pandemic’s long-term mental health outcomes from resulting infection, economic hardship, and other social stressors.^4,5^ After accounting for secular trends, there was no evidence of major disruption to depression screening processes, which likely benefitted from the VHA’s decades-long investment in Primary Care Mental Health Integration initiatives aimed at identifying, triaging, and initiating depression treatment directly in primary care.^1^ However, the COVID-19 pandemic and ensuing changes to clinic workflows impacted suicide risk screening directly.^3^ First, we posit that primary care teams, who administered most suicide risk screens, may be unfamiliar with C-SSRS and benefited from early implementation support, which may not have been sustained. Second, rapid, pandemic-driven expansion of telehealth care may have been affected by clinician concerns surrounding the ability to manage follow-up services for positive suicide risk.^6^ Our interrupted time series design is robust but limited to detecting population estimates and not mental health changes within individuals. Nevertheless, results support the need to strengthen mental health infrastructure (eg, suicide risk monitoring) in anticipation of increased demand from veterans and other patients at high risk.^5^ Future research should consider screening processes and assess whether this increase in positive screens corresponds to an increase in clinical depression diagnoses and suicide attempts or deaths and engagement in mental health treatment.

## Notes

Supplement.
Data Sharing Statement
