# Supplementary material for: Depression and Suicide Risk Screening in the Veterans Health Administration
Source: JAMA Netw Open. 2024 Dec 23;7(12):e2451936. doi: 10.1001/jamanetworkopen.2024.51936 (PMC11667358; doi:10.1001/jamanetworkopen.2024.51936)
Supplement: Supplement. — Data Sharing Statement [file jamanetwopen-e2451936-s001.pdf]

Data Sharing Statement

Leung. Depression and Suicide Risk Screening in the Veterans Health Administration. *JAMA Netw Open*. Published online December 23, 2024. doi:10.1001/jamanetworkopen.2024.51936

Data

Data available: No

Additional Information

Explanation for why data not available: Under the Health Insurance Portability and Accountability Act (HIPAA), the dataset used in this study cannot be shared publicly because it contains patient-level Protected Health Information/Personally Identifiable Information (PHI/PII) from the Veterans Health Administration. To gain access to this data, interested researchers must complete credentialing to conduct VA research, as well as data use agreements with the Primary Care Analytics Team (pcat@va.gov) and other relevant VHA data owners.
